# The efficacy of acupuncture-assisted blind insertion of bullet nasointestinal tube method in patients with ischemic stroke: A retrospective study

**DOI:** 10.12669/pjms.40.10.9936

**Published:** 2024-11

**Authors:** Hongyan Zhao, Jiayu Chen, Huan Chen, Xiaoxia Lv, Zhenchan Lu, Yanhua Sun

**Affiliations:** 1 Hongyan Zhao Department of Neurology, The Fifth Clinical School of Zhejiang University of Traditional Chinese Medicine, 1558 Sanhuan North Road, Huzhou, Zhejiang Province, P.R. China, 313000; 2 Jiayu Chen Department of Rehabilitation The Fifth Clinical School of Zhejiang University of Traditional Chinese Medicine, 1558 Sanhuan North Road, Huzhou, Zhejiang Province, P.R. China, 313000; 3 Huan Chen Department of Neurology, The Fifth Clinical School of Zhejiang University of Traditional Chinese Medicine, 1558 Sanhuan North Road, Huzhou, Zhejiang Province, P.R. China, 313000; 4 Xiaoxia Lv Department of Neurology, The Fifth Clinical School of Zhejiang University of Traditional Chinese Medicine, 1558 Sanhuan North Road, Huzhou, Zhejiang Province, P.R. China, 313000; 5 Zhenchan Lu Department of Neurology, The Fifth Clinical School of Zhejiang University of Traditional Chinese Medicine, 1558 Sanhuan North Road, Huzhou, Zhejiang Province, P.R. China, 313000; 6 Yanhua Sun Department of Rehabilitation The Fifth Clinical School of Zhejiang University of Traditional Chinese Medicine, 1558 Sanhuan North Road, Huzhou, Zhejiang Province, P.R. China, 313000

**Keywords:** Ischemic stroke, Traditional Chinese medicine acupuncture, Bullet nasointestinal tube, Blind insertion

## Abstract

**Objective:**

To explore the efficacy of Traditional Chinese Medicine (TCM) acupuncture-assisted blind insertion of bullet nasointestinal tube method in patients with ischemic stroke.

**Methods:**

This is a retrospective study using clinical records of 180 patients with ischemic stroke, hospitalized in the Department of Neurology of the Zhejiang Province Tertiary Class A Hospital, Huzhou, China from July 2022 to June 2023. The patients were divided into two groups on the basis of the nasogastric tube insertion method. We selected 90 patients for each group from the database of records by using a random list method. Patients in the observation group underwent TCM acupuncture-assisted blind insertion of nasointestinal tube and those in the control group underwent the traditional bedside blind insertion of bullet nasointestinal tube. Success rate of tube insertion, average tube insertion time, intragastric insertion time, stomach time to jejunal insertion, patient comfort, and patient satisfaction in both groups were compared.

**Results:**

The success rate of nasointestinal tube insertion was significantly higher in the observation group compared to the control group (96.6% and 88.9%, p<0.05). There was no statistically significant difference in the time of nasoenteral tube insertion into the stomach (p>0.05). The insertion time from stomach to jejunum in the observation group was significantly shorter than in the control group (p<0.05). The patient comfort and satisfaction levels were significantly higher in the observation group compared to the control group (p<0.05).

**Conclusion:**

TCM acupuncture-assisted blind insertion of bullet nasointestinal tube method improves the success rate of tube insertion, shortens the average catheterization time from the stomach to the jejunum and improves patient’s comfort and satisfaction.

## INTRODUCTION

Ischemic stroke is associated with high incidence, mortality, disability and recurrence rates.^1^ Ischemic stroke is a major public health problem and one of the leading causes of death worldwide.^2^ It is usually accompanied by dysphagia, which affects the nutritional intake of patients, and may lead to life-threatening complications such as aspiration pneumonia and suffocation.^3^ Therefore, current guidelines state that enteral nutritional support should be provided as early as possible for stroke patients with dysphagia to improve their prognosis.^4^,^5^ When the position of enteral nutrition preparation infusion is lowered (such as from the stomach to the proximal small intestine), the risk of reflux and aspiration can be significantly reduced.^6^ Retro-pyloric access can improve patients’ tolerance to enteral nutrition, accelerate the achievement of nutritional goals, and reduce the incidence of aspiration and pulmonary infections.^7^

However, the process of catheterization may be associated with some adverse reaction, and catheterization under the guidance of imaging equipment such as endoscopy or ultrasound is cumbersome.^8^-^10^ Stimulating Zusanli can increase gastrointestinal motility and relieve pain and sedation, reduce the patient’s fear, and improve the success rate of catheterization.^11^ Blind insertion of nasogastric tubes in critically ill patients after injection of metoclopramide at Zusanli acupoint can shorten the time of tube insertion, improve the success rate of tube insertion, and reduce the risk of adverse reactions.^12^ Therefore, methods that would improve the efficiency of catheterization process and reduce its negative impact on patient’s quality of life, are crucial. The concept of TCM acupuncture, also called “green therapy”. This stimulates the body’s own regulation of physiological functions, which ultimately allow the body to release its original substances for self-regulation. Thus, toxic damage can be avoided.^13^ Acupuncture and moxibustion of the corresponding acupoints can balance the nervous system and endocrine system of the body, thereby regulating gastrointestinal function and making the gastrointestinal tract move regularly. For example, Zusanli is a key acupoint for treating the digestive system, it strengthens the spleen and regulating the middle, eliminating accumulation and removing dampness; Zhongwan is a point for the stomach, it replenishes qi, relieving adverse qi and stopping vomiting; Neiguan can adjust the balance of the three energizers, and is a key acupoint for dredging meridians and calming the mind, harmonizing the stomach and relieving adverse qi, and harmonizing the stomach and stopping vomiting; Neiguan and Qihai both regulate gastrointestinal function by activating neurons in the nucleus tractus solitarius. Zusanli and Neiguan can promote gastric motility, while Zhongwan and Qihai can inhibit gastrointestinal hypermotility. Thus, acupuncture plays a role of two-way regulation, that is, no matter what pathological state the body is in, the effect of acupuncture can correct the body to develop in the direction of normal steady state.

To account for possible unfavorable factors during the catheterization process, the Neurology Department of the Zhejiang Province Tertiary Class A Hospital, Huzhou, China in collaboration with the Rehabilitation Department, implemented TCM acupuncture in combination with nasointestinal catheterization in treating patients with ischemic stroke. The aim of this retrospective study was to evaluate the effectiveness of combining TCM acupuncture of catheterization using blindly inserted bullet nasointestinal tubes in stroke patients.

## METHODS

Clinical records of 180 patients with ischemic stroke and moderate to severe dysphagia, who were hospitalized in the Department of Neurology of our hospital from July 2022 to June 2023, were retrospectively selected. Patients were grouped into an observation and a control group based on the method used for bullet nasointestinal tube insertion, there were 90 patients in each group.

### Ethical Approval:

It was obtained from Medical Ethics Committee of Huzhou Central Hospital via approval number: 202112003-01, dated December 16^th^, 2021.

### Inclusion criteria:

- Patients meeting the diagnostic criteria for cerebral infarction and cerebral hemorrhage according to the “Key Points for Diagnosis of Various Major Cerebrovascular Diseases in China 2019”.^14^
- Age between 18 and 80 years.
- First stroke.
- The severity of dysphagia screened by Kubota^15^ screening drinking water test reaching a level between III and V.
- The patient’s vital signs are stable.

### Exclusion criteria:

- Patients with oral diseases or mucosal damage.
- Patients with tracheotomy.
- Patients whose state of consciousness has changed and are unable to cooperate.
- Patients with swallowing dysfunction caused by other diseases.

Baseline characteristics of patients were collected, including the Acute Physiology and Chronic Health Evaluation System (APACHE II)^16^ scores, Neurological Impairment Scores (NIHSS),^17^ and Kubota drinking water test results. All patients underwent evaluation by the nutritional support team to confirm that they meet the requirement for the enteral nutrition, justifying the indication for blind bedside catheterization. Informed and signed consent was taken from the family members prior procedure. They were counselled and fully informed about the procedure, advantages and disadvantages of catheterization.

The patients, selected by using a random list method from the database of records, were divided into two different groups with 90 cases in each. Catheterization in both groups of patients was done using the Jiancheng NTIII-12 gravity cranio-nasointestinal tube, also known as bullet nasointestinal tube. The operator confirmed that the tube has reached the stomach by listening to the sound of air passing through water or by withdrawing gastric juice.

For patients in the control group, catheterization was performed using the traditional bedside blind nasointestinal tube insertion method, with the patient in a sitting or semi-sitting position. The steps were the same as for the gastric tube insertion. The tube was inserted 70cm into the stomach, and then the advancement speed was reduced. During the intubation process, the rotational advancement method was used to continue the intubation to 90-105cm, and then X-ray was used to confirm the catheter position.

For patients in the observation group, the acupuncturist performed acupuncture on both sides of Zusanli, Neiguan, Zhongwan and Qihai to promote gastrointestinal peristalsis. During the catheterization period, the acupuncturist fine-tuned the stimulation site through manual manipulation according to the speed of intestinal entry. The operating nurse changed the catheterization technique after inserting the catheter into the stomach and used the index finger pressure method. Briefly, the catheter was coiled into a circle and the tail end was fixed in the left hand of the operator. The catheter was bent into a semicircle with the right hand, 5cm away from the tip of the nose.

The index finger of the right hand was used to continuously and evenly apply 25-30cmH_2_O pressure to the nasointestinal tube (with the pressure similar to touching the tip of the nose). The inward speed and frequency of the tube depended on the patient’s breathing rhythm and frequency. Every time the patient inhaled, the tube was advanced about 1cm. Sudden resistance indicated that the pylorus was reached. The operator continued to slowly advance the tube inward until the measured length was reached. Next, air injection and auscultation of the intestinal tract was performed. The operator used a 50 ml empty needle to quickly inject 20 ml of air into the tube.

At the same time, a stethoscope was used to auscultate the patient’s left upper abdomen, right abdomen, periumbilical area, and left abdomen in sequence, and to regulate the air flow through the intestines at a high speed. The sound was used to determine the position of the catheter. The high-pitched sound of air passing through fluid on the right side of the abdomen, around the umbilicus, or on the left side of the abdomen indicated that the catheter has passed through the pylorus. A plain abdominal X-ray was done to confirm the position. The following observation indicators were collected:

### Total catheterization success rate:

The catheterization success rate was defined as the ratio of the number of successful catheterization cases to the total number of catheterization cases, and the final success of catheterization after accurately judging that the catheter tip is located in the pylorus through X-ray examination.

### Success rate of primary catheterization:

A procedure was deemed successful if the tip of the nasointestinal tube entered the horizontal part of the duodenum, as confirmed by the abdominal X-ray.

The time of catheterization to the pylorus was noted. In addition, patient comfort was assessed by the Visual Analogue Scale (VAS).^18^ The scale can be used to assess the patient’s comfort level using a score from 0 to 10, with 0 being very comfortable and 10 being extremely uncomfortable. Patients are guided through the testing by nursing staff from the nutritional management team. Score of 0 to two points was classified as comfort; three to four points- mild discomfort; five to six points- moderate discomfort; 7 to 8 points- severe discomfort; and 9 to 10 points- as extreme discomfort. The patients were instructed or assisted by a nursing staff from the nutrition management team during the completion of the test.

Patient satisfaction with enteral nutrition nursing work was assessed using a questionnaire based on the evaluation scale by the National Health Commission’s “Enteral Nutrition Nursing Demonstration Ward Evaluation Index System and Evaluation Criteria”. The questionnaire included a total of 14 items, divided into service attitudes (four items), technical level (one item), professional quality (four items), health education (four items) and comprehensive evaluation (one item). The scoring method was as follows: “five points” - Very satisfied, “four points” - satisfied, “three points” - average, “two points” - dissatisfied, and “one point” - very dissatisfied. The highest score of this scale was 70 points, and the lowest score was 14 points. The higher the total score, the more satisfied the inpatients were with enteral nutrition care. This study used a percentage system for conversion. The patients were instructed or assisted by nursing, staff from the nutrition management team during the completion of the test.

### Statistical analysis:

SPSS22.0 statistical software was used for data analysis. The categorical data were described by the number of cases and composition ratio (%) and analyzed by the χ^2^ test. The quantitative data consistent with the normal distribution were described by the mean ± standard deviation (χ̅±*S*), with two rows. Independent sample t test analysis was done. A p-Value of <0.05 was considered statistically significant.

## RESULTS

There was no statistically significant difference (p>0.05) in the age, gender, APACHE-II score, NIHSS, and Kubota drinking water test between the two groups of patients (Table-I).

**Table-I:** Comparison of the general information of the two groups of patients.

Patient information	Observation group (n = 90)	Control group (n = 90)	x^2^/t	p-Value
Gender (male/female, n)	43/47	49/41	0.796	0.372
Age	58.13±13.41	56.35±15.01	0.839	0.403
BMI (kg/m^2^)	22.31±4.15	22.15±4.46	0.249	0.803
APACHE II score	17.55±8.15	16.91±8.47	0.517	0.606
NIHSS score	6.55±4.81	6.31±4.98	0.329	0.742

### Catheterization success rates comparison:

In this study, there were 13 cases (six cases in the observation group and seven cases in the control group) of failed intragastric catheter insertion. The success rate of intragastric catheterization was 93.3%. In the observation group, and 92.2% in the control group. There were 13 cases of failed nasojejunal tube placement. Of them, three cases were in the observation group and 10 cases in the control group. The success rate of nasojejunal tube insertion was 96.6% in the observation group and 88.9% in the control group. There was no statistically significant difference between the two groups in the success rates of intragastric placement (p>0.05), the difference of the success rate of nasojejunal was statistically significant (p<0.05). Table-II. Comparison of the insertion time into the stomach and the insertion time from the stomach to the jejunum. There was no statistically significant difference in the intragastric insertion time between the two groups (p>0.05). As shown in Table-III, the time from stomach to post-pylorus insertion in the control group was significantly shorter than that in the control group (p<0.01).

**Table-II:** Comparison of the success rates of intragastric and gastric to nasojejunal tube placing between the two groups (%).

Group	Number of examples	Gastric insertion	After inserting the pylorus from the stomach
Observation group	90	93.3(84/90)	96.6(87/90)
Control group	90	92.2(83/90)	88.9(80/90)
X^2^ value	-	0.083	4.040
p-Value	-	0.774	0.044

**Table-III:** Comparison of the insertion time in the stomach and the insertion time from the stomach to the jejunum between the two groups (minχ̅±*S*)

Group	Insertion time in stomach	Time from stomach to post-pylorus
Observation group	2.32±0.58	12.35±5.01
Control group	2.26±0.47	22.56±7.56
t value	0.762	10.680
p-Value	0.447	<0.001

### Patient comfort and satisfaction:

Patient satisfaction and the patient comfort level in the observation group were significantly higher than that in the control group (p<0.05; Table-IV)

**Table-IV:** Patient satisfaction and comfort scores of the two groups (points,χ̅±*S*).

Group	Number of examples	Satisfaction score	Comfort score
Observation group	90	94.32±4.71	91.74±3.34
Control group	90	84.79±9.26	82.49±7.56
t value	-	8.702	10.618
p-Value	-	<0.0001	<0.0001

## DISCUSSION

This study retrospectively assessed the efficiency of TCM acupuncture combined with the blind insertion of bullet nasointestinal tube method of nasointestinal catheterization. Our results showed that the TCM acupuncture-assisted method was associated with shorter time from stomach to post-pylorus insertion, and improved patient satisfaction and comfort level compared to traditional nasointestinal catheterization.

Jianhua W et al.^19^ compared acupuncture with metoclopramide and found that acupuncture can improve the success rate of catheterization and shorten the catheterization time. Acupuncture can also reduce the occurrence of reflux during the catheterization process. Although there was no significant difference between the two groups in terms of pulmonary infection, the pulmonary infection rate in the acupuncture group was lower than that in the control group, suggesting that acupuncture may reduce the occurrence of pneumonia. This study is different from the study by Lingwei Y et al.,^20^ who combined acupuncture with moxibustion, but both proved the effectiveness of acupuncture in increasing the rate of catheterization. Aiping B et al.^21^ compared acupuncture with domperidone and found that the success rate of catheter placement in the acupuncture group was better than that of the domperidone group. In the same study, acupuncture reduced the incidence of diarrhea and increased the level of nutritional indicator serum albumin. It can be seen from multiple studies that acupuncture therapy is simple, safe, efficient, and superior to gastric motility drug therapy.

Malnutrition or increased nutritional risks prolong the hospitalization time of stroke patients, increase the risk of post-stroke disability and mortality, and bring huge physical, mental and economic burdens to patients, their families, and healthcare system.^22^-^24^ Therefore, providing patients with reasonable and effective nutritional support is one of the current key clinical concerns. Blind nasointestinal tube insertion is one of the common methods for early enteral nutrition in patients with ischemic stroke. This method has the advantages of convenience, non-invasiveness, and low cost, but it requires clinical experience of the operator. High skill requirements may delay the implementation of gastrointestinal nutrition support treatment.^25^,^26^

The success rate of blind insertion has always been lower than that of interventional endoscopic contrast agent- and X-ray guided tube insertion. In recent years, clinical placement of nasoenteric tubes is often done with the help of endoscopic tube insertion. Since these methods require certain technical and pharmaceutical support, their clinical application is limited and there are unavoidable adverse reactions. TCM acupuncture stimulates the patient’s own regulation of physiological functions without adding additional new substances, allowing body to release its original substances for self-regulation, and thereby avoiding toxic damage.

The results of our study showed that there was no statistical significance in the gender, age, BMI, and APACHE-II scores of the two groups of patients (p>0.05). Similarly, success rates of catheterization between the two groups were comparable. The insertion time from the stomach to the pylorus was significantly shorter in the observation group. We may speculate that acupuncture at Zusanli and Neiguan promotes regular peristalsis of the gastrointestinal tract. Patient satisfaction and comfort levels of the observation group were significantly higher compared to the control group, and the difference between the two groups was statistically significant (p<0.05. This effect may be related to the fact that the combined method can reduce the occurrence of reflux, nausea, vomiting, etc., improve the success rate of catheterization, and shorten the overall catheterization time.

### Limitations:

It is a retrospective single-center study with a small sample size. Additionally, the study was limited to intensive care unit setting. Therefore, our evidence is insufficient to support clinical promotion of the method. Further multi-center large studies are needed to assess whether TCM methods may be integrated into modern medical and nursing technologies.

## CONCLUSION

We have showed that TCM acupuncture-assisted blind insertion of nasointestinal tube method is simple, safe and efficient for inpatients with ischemic stroke. It is associated with short intubation time, good patient tolerance, high intubation success rate, and improves patient’s comfort and satisfaction levels.

### Authors’ contributions:

**HZ:** Conceived, designed the study and Review.

**HZ**, **JC**, **HC**, **XL**, **ZL** and **YS:** Collected the data, performed the analysis, critical review, were involved in the writing of the manuscript

All authors have read and approved the final manuscript and are responsible for the integrity of the study.
